# Identification of Perioperative Risk Factors for Early Sacral Nerve Stimulator Explantation: A Single-Center Retrospective Cohort Study

**DOI:** 10.3390/jcm14072363

**Published:** 2025-03-29

**Authors:** Peyton J. Murin, Patrick J. Murin, Yara Lima de Mendonça, Yuri Chaves Martins

**Affiliations:** 1 Department of Neurology, Saint Louis University School of Medicine, St. Louis, MO 63104, USA; peyton.murin@slucare.ssmhealth.com; 2 Department of Psychology, Rhodes College, Memphis, TN 38112, USA; murpj-27@rhodes.edu; 3 Lower Gastrointestinal Surgery Department, The Royal Marsden Hospital, London SW3 6JJ, UK; yara.limademendonca@rmh.nhs.uk; 4 Department of Anesthesiology, Saint Louis University School of Medicine, St. Louis, MO 63110, USA

**Keywords:** sacral nerve stimulator, chronic pain management, explantation surgery, surgical indication, urinary incontinence

## Abstract

**Background:** Sacral nerve stimulators (SNSs) can be an effective treatment for urinary incontinence. However, with a failure rate of up to 50%, an explantation rate of up to 16%, and a cost of ~USD 10,000 per implant, identification of patients at high risk for explantation is necessary to improve patient satisfaction and reduce the economic burden on the healthcare system. The objective of this retrospective cohort study was to determine predictors of SNS explantation within the first two years of device placement. **Methods:** The MOVER database was queried for patients with a SNS and at least two years of follow-up (n = 54). Multivariate logistic regression was performed to assess risk factors for explantation. Factor optimization was used to eliminate factors with limited predictive value. **Results:** The model displayed excellent performance with an AUC of 0.93 (95% CI: 0.78–1.00) and an f1-score of 0.81. Female sex (OR: 3.75; CI: 3.71–3.79), malignancy (OR: 3.14; CI: 3.10–3.18), ASA score (OR: 2.53; CI: 2.50–2.57), peripheral neuropathy (OR: 2.04: CI: 2.01–2.07), alcohol use (OR: 1.98; CI: 1.96–2.01), and length of stay (OR: 1.47; CI: 1.45–1.49) displayed statistically significant increased risk of explantation. Atrial fibrillation (OR: 0.36; CI: 0.35–0.36) and chronic kidney disease (OR: 0.54; CI: 0.53–0.54) were included in the model but conferred decreased risk of explantation. **Conclusions:** Patient ASA score and a medical history of malignancy, peripheral neuropathy, and alcohol use are possible novel risk factors for SNS explantation.

## 1. Introduction

Urinary incontinence is a prevalent condition [1,2] with a complex pathophysiology involving the central and peripheral nervous systems [3]. Sacral nerve stimulation (SNS) is a treatment option used to alleviate urinary incontinence, chronic pelvic pain, and constipation in patients unresponsive to more conservative therapies [4]. SNS is generally considered safe and effective when used correctly in carefully selected patients with various conditions including urgency incontinence [5], idiopathic urinary incontinence [6], and neurogenic lower urinary tract dysfunction [7]. However, device failure and explant continue to be important problems [8,9]. Long-term response to SNS remains unacceptably low with a failure rate of up to 50% and an explantation rate of up to 16% [8,9]. With a total implant cost estimated to be over USD 10,000 [10], early explantation of SNS poses a significant economic burden to patients and the healthcare system. Key reasons for removal include poor SNS efficacy, pain, infection, and facilitation of magnetic resonance imaging scans [9].

Unfortunately, identification of factors associated with SNS explant remains challenging [11]. Previous work seeking to identify risk factors for explantation has identified age less than 55 as predictive of explanation [12]. However, efforts to identify other demographic, medical, social, or behavioral factors predictive of explantation have been unsuccessful [12,13]. To address this challenge, we sought to apply machine learning in the form of a multivariate logistic regression to identify demographic, medical, social, or behavioral factors predictive of early sacral nerve stimulator explantation—defined as explantation within two years of device placement.

## 2. Materials and Methods

This study was conducted in accordance with TRIPOD-AI guidelines for reporting clinical prediction models that use regression or machine learning methods.

Data: We used the Medical Informatics Operating room Vitals and Events Repository (MOVER) dataset [14], which contained electronic health records (EHRs) from 58,799 unique patients undergoing surgery at the University of California Irvine Medical Center (UCI) between January 2018 and July 2023. Data included in the MOVER database were compiled using a retrospective review of the EHRs and waveform matching. Data were de-identified according to HIPAA Privacy Rule standards, with patient health information (PHI) manually removed from free text, age capped at 90, and dates shifted by a consistent, random number of days to further ensure patient anonymity. As data were de-identified according to the HIPAA Privacy Rule, patient consent was not required. To access the data, a data use agreement (DUA) was initiated between the authors and UCI. This study was reviewed by the Saint Louis University institutional review board (IRB) and determined to be exempt from review. Data included within the MOVER database consist of comprehensive EHR data complete with patient demographics, medical comorbidities, surgical procedure, and operative medicines, lines, and drains, and any postoperative complications. The dataset is maintained and updated by the study team at UCI.

**Figure 1:**
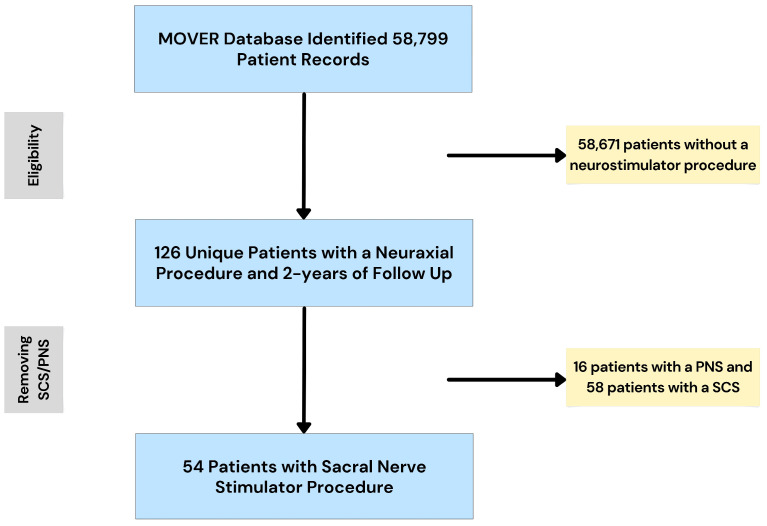
Flowchart demonstrating the sampling process. The MOVER database contained 58,799 unique patient records, from which we identified 126 unique patients with a neuraxial procedure and at least 2 years of follow-up. Of these 126 patients, 54 had a sacral nerve stimulator. SCS: spinal cord stimulator; PNS: peripheral nerve stimulator.

Participants: The MOVER database included participants undergoing surgery at a single center (UCI). Treatments received consisted of sacral nerve stimulators. Inclusion criteria: adult (≥18 years of age), SNS procedure, and ≥2 years of follow-up. Exclusion criteria: <18 years of age, <2 years of follow-up, peripheral nerve stimulator (n = 16) and spinal cord stimulator (n = 58). The overall study cohort consisted of 54 unique patients with SNS and at least 2-years of follow-up. Figure 1 illustrates the sampling process.

To assess the representativeness of our sample, we compared the demographic and clinical characteristics of included patients to previous studies [15]. Our cohort had a higher proportion of female patients and an average age greater than 60 years, which aligns with prior studies on SNS recipients [16]. However, our cohort mainly focused on patients with urinary dysfunction, and patients with other indications for SNS placement such as fecal incontinence were poorly or not represented.

Data Preparation: All study variables were created from data elements in the MOVER dataset. The International Classification of Diseases (ICD)-9-CM, procedure codes/claims (ICD-9, -10, and CPT), demographic data, American Society of Anesthesiologist (ASA) score, anesthesia type, and postoperative events including hospitalizations, intensive care unit (ICU) admissions, and demographics were used to create study variables. Detailed definitions of all study variables are available in Table S1. Sex was encoded 1: female, 0: male. Medical comorbidities were encoded 1: present, 0: absent. Anesthesia type was encoded 1: monitored anesthesia care, 0: general anesthesia. ICU admission was encoded 1: yes, 0: no. LOS and ASA score was encoded as the numerical value.

Outcome: The primary outcome of our study was early SNS explantation. Early explantation was defined as having surgery for removal of SNS due to any reason in the first two years post-implantation.

Predictors: Study variables included a broad range of demographic, medical comorbidity, psychiatric comorbidity, and perioperative variables based upon available data and the prior literature. As the statistical plan included recursive factor elimination, the model would remove variables offering limited predictive value, so we did not attempt to limit variables at this stage.

Sample Size: All patients meeting inclusion/exclusion criteria were included.

Missing Data: No missing data were encountered in this study.

**Figure 2:**
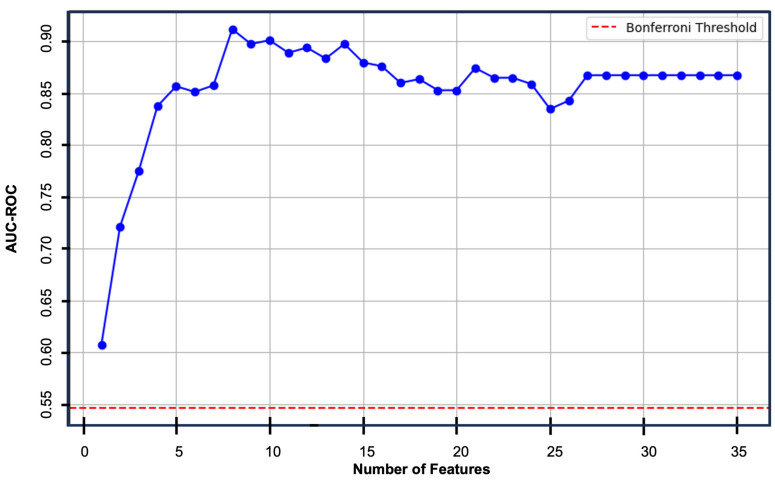
Recursive feature elimination. The logistic regression algorithm was instructed to loop through, removing the least predictive feature with each iteration, until the optimal number of features was identified based upon the area under the receiver operating characteristic curve (AUC-ROC). The blue line represents the AUC-ROC value at each respective number of variables. Eight features resulted in the optimal AUC-ROC of 0.93. The red-dotted line represents the Bonferroni threshold for statistical significance.

Analytical Methods: The data analysis plan was written before accessing the data. Numerical variables were reported as means ± standard deviation (SD) or medians with interquartile range (IQR). Categorical variables were reported as proportions or percentages. As an initial step, we compared patients with early explantation to those without early explantation using Fisher’s exact test for nominal variables and the Mann–Whitney U test for ordinal or continuous variables. Statistical significance was set at *p* < 0.05. To account for the interactions between variables, we trained and fitting a multivariable logistic regression. Data were imported into Anaconda Version 2.3.1. (Anaconda Software Distribution, Austin, TX, USA). The following add-ons were used for analysis: sklearn23 [17], MATLAB (The MathWorks, Inc., Natick, MA, USA), pandas24 [18], scikit-learn-extra [17], and seaborn [19]. The target variable (explantation or no explantation) was defined, the features were scaled, and the Synthetic Minority Oversampling Technique (SMOTE) was applied to balance the classes. The data were split into training (80%) and testing (20%) sets using random state to create deterministic train–test sets and the multivariate logistic regression model was trained. The model was then used to make predictions, and performance was evaluated using a train–test approach with reporting of precision, recall, f1-score, and area under the receiver operating curve (AUC-ROC). As a next step, recursive feature elimination (RFE) was imported from sklearn.feature_selection and cross_val_score was imported from sklearn.model_selection. The algorithm iteratively evaluated different combinations of variables to identify the optimal set based on the AUC-ROC with cross-validation (Figure 2). Bonferroni correction was performed with Bonferroni-corrected significance level of 0.0014.

The optimal number of features was identified, and logistic regression was repeated as above with optimal number of features. Again, performance was measured using precision, recall, f1-score, and AUC-ROC. As a secondary measure, bootstrap validation was performed, and a calibration curve was created. Once performance was assessed, the model was instructed to calculate odds ratios and 95% confidence intervals. The odds ratio was considered statistically significant (*p* < 0.05) if the entire 95% confidence was greater than 1 (increased risk) or less than 1 (decreased risk).

Class Imbalance: Class imbalance was addressed using the SMOTE.

Fairness: Model fairness was assessed using recursive factor elimination with cross-validation, calibration curve, and bootstrap validation to minimize overfitting and most accurately report model performance.

Model Output: Model output consisted of an odds ratio and standard deviation. For rare outcomes, the odds ratio approximates relative risk. Threshold for significance was an odds ratio and 95% confidence interval >1.0.

## 3. Results

### 3.1. Cohort Demographics

Baseline cohort characteristics are shown in Table 1. The overall cohort was 87.0% female with an average age of 63.7 (+/−15.9) years. Urinary dysfunction was the most common indication, comprising 98.1% of patients. The average score ASA was 3.0 (IQR: 2–3), and the cases were predominantly done using monitored anesthesia care (72.2%). The average length of stay following implantation was 0.1 (+/−0.5) days, with no statistically significant difference in length of stay in patients with explanation compared to those without explantation. The most common medical comorbidities were musculoskeletal pain (27.8%), sleep disorders (24.1%), arthritis (20.4%), and hypertension (16.7%). Hyperlipidemia was more frequently seen in patients without explantation (31.4%) compared to those with explantation (7.9%). No other statistically significant differences were seen between the cohorts.

### 3.2. Multivariate Logistic Regression Model

**Figure 3:**
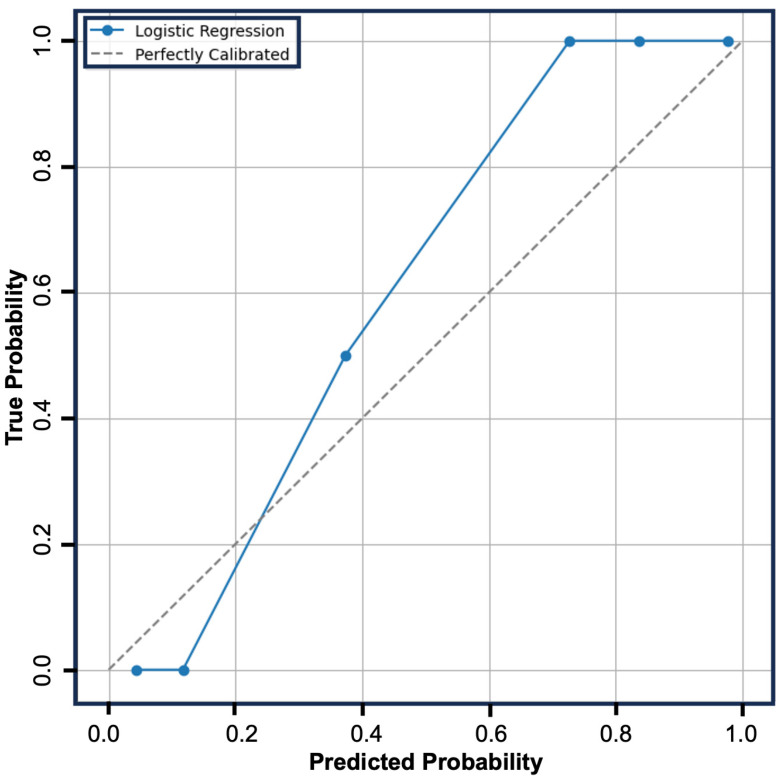
Calibration curve providing a visual assessment of the logistic regression model performance. The grey dotted line represents a perfectly calibrated model. The blue solid line represents the model’s performance. The model displayed robust performance with an AUC: 0.93.

**Figure 4:**
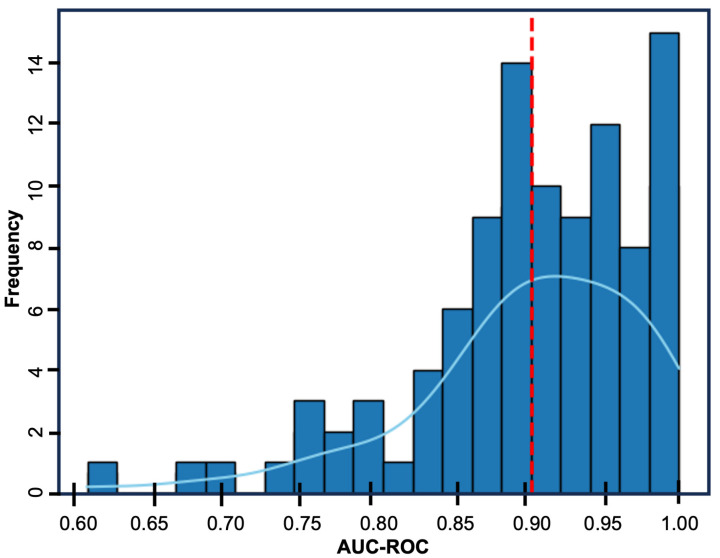
Bar graph providing a visual representation of the bootstrap validation. The model was repeated 100 times, with the bars representing the frequency of each AUC-ROC value. The red-dotted line represents the mean AUC-ROC and the light blue line represents the line of best fit. The model displayed an average AUC-ROC of 0.93, with a 95% confidence interval of 0.78 to 1.00.

Given the limited associations identified by basic statistical analysis, we hypothesized an effective predictive model would need to be able to account for the interactions between multiple variables. To this end, we applied supervised machine learning in the form of a multivariable logistic regression model that allows for robust assessment of between variable interactions. The initial logistic regression model displayed good performance with an average precision of 0.81, an average recall of 0.69, and average f1-score of 0.65, and an average AUC-ROC of 0.86. Feature optimization identified nine features, sex, ASA score, length of stay, peripheral neuropathy, low back pain, atrial fibrillation, chronic kidney disease, malignancy, and alcohol use as most predictive. Repeating the logistic regression analysis with these nine features, the model displayed excellent performance with an average precision of 0.86, an average recall of 0.81, an average f1-score of 0.81, and an average AUC-ROC of 0.93 (95% CI: 0.7795–1.0000). (Figure 3: calibration curve) (Figure 4: bootstrap AUC-ROC)

**Figure 5:**
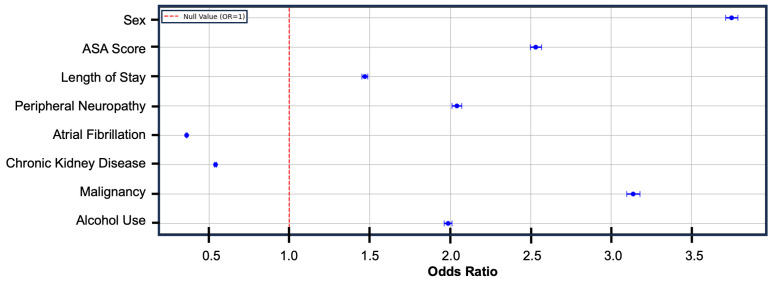
Forest plot demonstrating female sex, malignancy, higher ASA score, peripheral neuropathy, alcohol use, and length of stay following implant as predictive of increased risk of SNS explantation. Atrial fibrillation and chronic kidney disease were predictive of decreased risk. The dot represents the odds ratio, with the brackets indicating the 95% confidence interval. The red dotted line represents an odds ratio of 1.0.

Amongst the assessed variables, six were predictive of increased risk of explantation. They were female sex (OR: 3.75; CI: 3.71–3.79), malignancy (OR: 3.14; CI: 3.10–3.18), ASA score (OR: 2.53; CI: 2.50–2.57), peripheral neuropathy (OR: 2.04: CI: 2.01–2.07), alcohol use (OR: 1.98; CI: 1.96–2.01), and length of stay (OR: 1.47; CI: 1.45–1.49). Atrial fibrillation (OR: 0.36; CI: 0.35–0.36) and chronic kidney disease (OR: 0.54; CI: 0.53–0.54) were included in the model; however, both conferred decreased risk of explantation (Figure 5; Table S2).

## 4. Discussion

SNS is an effective therapeutic option for multiple types of urinary incontinence [5,6,7]. However, targeting therapy to the correct patients remains challenging, with an explantation rate of 5–16% [8,12], resulting in significant expense to patients and the healthcare system [10]. Here, we applied supervised machine learning in the form of multivariate logistic regression to a large publicly available database seeking to identify factors predictive of SNS explantation. Utilizing multivariate logistic regression combined with feature optimization, we were able to create a model using eight key patient factors that was able to predict explantation with an average AUC-ROC of 0.93. In doing so, we identified six factors predictive of increased risk of SNS explantation (female sex, malignancy, higher ASA score, peripheral neuropathy, alcohol use, and length of stay following implant).

Evaluating the factors selected for the model and their clinical utility, there are several considerations. To begin, the length of stay following the procedure could be reflective of the procedure itself and, therefore, may not be a modifiable risk factor. Furthermore, while certain factors (alcohol use, length of stay, and chronic kidney disease) were considered statistically significant, their relative clinical utility could be limited given their proximity to an odds ratio of 1.0. The finding of atrial fibrillation was unexpected. It has been previously shown that patients with atrial fibrillation undergoing noncardiac procedures are at higher risk of morbidity and mortality [20]. However, due to this increased risk of morbidity and mortality, these patients are typically subjected to more robust preoperative risk stratification and evaluation [21]. It is plausible, this more robust preoperative evaluation combined with the elective nature of the SCS implant procedure results in only low-risk atrial fibrillation patient undergoing the procedure.

We are not the first to attempt to address this challenge. A single-center retrospective cohort study used a logistic regression approach seeking to identify patients factors predictive of SNS explantation. While they did identify age under 55 years as predictive of explantation or revision, they were unable to identify further associations with other demographic factors or medical comorbidities [12]. A second retrospective study sought to identify risk factors for battery explantation in a Medicare database. While they were able to identify interstitial cystitis as predictive of explantation, again, no other associations between demographic factors or medical comorbidities were identified [22]. Another related study sought to identify factors predictive of SNS infection, which may necessitate explantation or revision. The authors identified postoperative hematoma and a deep pocket (>3 cm) as predictive of infection within 180 days following implantation [23].

In comparison to previous attempts, our approach achieved significantly higher performance and identified six novel patient factors predictive of explantation. We propose the reason for this efficacy lies in the utilization of factor optimization. Given the paucity of literature on risk factors for SNS explantation [12,22], we started our analysis using a broad battery of demographic, preoperative, and perioperative factors. With all factors included, the logistic regression model demonstrated average performance (AUC of 0.86) with poor precision (average of 0.69). We hypothesized this was due to many factors offering limited predictive value. To optimize the model systematically and objectively, we performed factor optimization using an approach removing the least predictive factor until an optimal AUC was achieved. This resulted in improved overall performance (AUC: 0.93) and improved precision (average precision: 0.86), while simultaneously narrowing the number of assessed factors to eight total factors, with six factors conveying increased risk of explantation.

Looking to the future, it is necessary to consider how to incorporate these findings into the preoperative evaluation. As an initial step, we recommend that the four medical comorbidity risk factors be considered in the decision to pursue SNS. When possible, this should include a multidisciplinary collaboration with relevant specialists to best qualify the potential risk conferred by these conditions and how to best mitigate the risk (if possible). In malignancy or peripheral neuropathy, for example, it is relevant to consider the level of control of the comorbid condition. If poorly controlled, such factors are likely to impact effectiveness and tolerance of SNS. In patients with multiple risk factors (e.g., female sex + malignancy) or poorly controlled risk factors, we recommend caution in pursuing SNS. In cases where SNS is pursued, we recommend a robust discussion of risk of explant in the preoperative risk–benefit discussion. When the decision is made to pursue the procedure in ‘high-risk’ patients, it is imperative for robust postoperative follow-up. At baseline, SNS confers a risk of explant of 5–16% [8,9]. Our work suggests that in patients with the above-described risk factors this risk is likely significantly higher. As such, it is important to monitor these patients closely to allow for early identification of device dysfunction, device infection, or other complications, and mitigation of resultant morbidity.

Strengths of our study include the robust number of demographic, medical history, perioperative, and behavioral factors assessed; the use of supervised machine learning; the use of factor optimization; and the robust follow-up period. There were multiple limitations to our study, many of which are inherent to retrospective database research. We were unable to perform detailed chart reviews to verify the accuracy of the database information. This is a single-center study with a relatively small number of patients included (n = 54). Thus, results may not generalize to other centers. In addition, like with any observational study, we also cannot exclude the possibility that residual confounding could have biased our results. Ideally, we would have had external data to validate the model, but due to the lack of such data, this was not possible at this time. Further limitations include the inherent risk of overfitting in a small dataset and rare outcome. While we took many steps to mitigate this risk (recursive factors elimination with cross validation, bootstrap validation, and visualizing performance with a calibration curve), it is not possible to entirely mitigate this limitation.

There are multiple avenues for future study. Future prospective studies are needed to validate the effectiveness of the model in predicting explant risk. While the model was applied to SNS explant, our statistical framework is conducive to other medical dilemmas. We plan to utilize the framework and apply it to evaluate risk for other neuromodulation procedures (e.g., deep brain stimulation, spinal cord stimulator); however, it would offer potential utility in assessing any rare postoperative complication. Additionally, further studies are indicated to elucidate the clinical and pathophysiological basis for the increased risk of explant conferred by medical comorbidities.

## Figures and Tables

**Table 1:** Baseline cohort characteristics.

Variable	Total	Explant	No Explant	*p* Value
Number of patients	54	38	16	
Sex				
Male	7 (13.0%)	2 (5.3%)	4 (25.0%)	0.0563
Female	47 (87.0%)	36 (94.7%)	12 (75.0%)	0.0563
Age (years ± SD) ^1^	63.7 ± 15.9	63.8 ± 16.1	63.3 ± 15.8	0.8548
ASA score (IQR) ^1^	3 (2–3)	3 (2–3)	2 (2–3)	0.1608
Anesthesia type				
Monitored anesthesia care	39 (72.2%)	27 (71.1%)	12 (75.0%)	>0.9999
General anesthesia	15 (27.8%)	11 (28.9%)	4 (25.0%)	>0.9999
Length of stay (days ± SD) ^1^	0.1 ± 0.5	0.2 ± 0.5	0.0 ± 0.0	0.5465
Indications for SNS ^2^				
Peripheral neuropathy	6 (11.1%)	5 (13.2%)	1 (6.3%)	0.6570
Low back pain	6 (11.1%)	4 (10.5%)	2 (12.5%)	>0.9999
Cervical pain	3 (5.6%)	2 (5.3%)	1 (6.3%)	>0.9999
Urinary dysfunction	53 (98.1%)	37 (97.4%)	16 (100.0%)	>0.9999
Past Medical History				
Cerebrovascular disease	1 (2.0%)	0 (0.0%)	1 (6.3%)	0.2963
Obstructive sleep apnea	9 (16.7%)	4 (10.5%)	5 (31.3%)	0.1056
Sleep disorder	13 (24.1%)	8 (21.1%)	5 (31.3%)	0.4934
Hypertension	9 (16.7%)	6 (15.8%)	3 (18.8%)	>0.9999
Hyperlipidemia	8 (14.8%)	3 (7.9%)	5 (31.4%)	0.0413 *
Atrial fibrillation	3 (5.6%)	1 (2.6%)	2 (12.5%)	0.2064
Diabetes mellitus	5 (9.3%)	3 (7.9%)	2 (12.5%)	0.6265
Chronic kidney disease	1 (1.9%)	0 (0.0%)	1 (6.3%)	0.2963
Anxiety	9 (16.7%)	7 (18.4%)	2 (12.5%)	0.7093
Depression	5 (9.3%)	5 (13.2%)	0 (0.0%)	0.3064
Obesity	3 (5.6%)	2 (5.3%)	1 (6.3%)	>0.9999
Migraine	4 (7.4%)	4 (10.5%)	0 (0.0%)	0.3064
Musculoskeletal pain	15 (27.8%)	9 (23.7%)	6 (37.5%)	0.3332
Arthritis	11 (20.4%)	8 (21.1%)	3 (18.8%)	>0.9999
Malignancy	8 (14.8%)	8 (21.1%)	0 (0.0%)	0.0883
Social History				
Opioid use disorder	1 (1.9%)	1 (2.6%)	0 (0.0%)	>0.9999
Illicit substance use	1 (1.9%)	1 (2.6%)	0 (0.0%)	>0.9999
Alcohol use	1 (1.9%)	1 (2.6%)	0 (0.0%)	>0.9999
Tobacco products	2 (3.7%)	0 (0.0%)	2 (12.5%)	0.0839

^1^ Numerical variables were reported as means ± standard deviation (SD) or medians with interquartile range (IQR). ^2^ In patients with multiple possible indications for neuromodulation, they were included in both groups. Categorical variables were reported as n and percentages (%). * = *p* < 0.05.

## Data Availability

The data that support the findings of this study are openly available in the MOVER database at https://mover.ics.uci.edu/index.html (accessed 17 May 2024), reference [14].

## Supplementary Materials

The following supporting information can be downloaded at: https://www.mdpi.com/article/10.3390/jcm14072363/s1, Table S1: Detailed definitions of all study variables; Table S2: Odds ratio, 95% confidence interval, and *p*-value of multivariate logistic regression after feature optimization.

## Abbreviations

The following abbreviations are used in this manuscript:

ASA	American Society of Anesthesiologists
AUC	area under the curve
CI	confidence interval
CKD	chronic kidney disease
EHR	electronic health records
ICD	International Classification of Diseases
ICU	intensive care unit
IQR	interquartile range
MOVER	Medical Informatics Operating room Vitals and Events Repository
OR	odds ratio
ROC	receiver operating characteristic
SCS	spinal cord stimulator
SD	standard deviation
SMOTE	Synthetic Minority Oversampling Technique
SNS	sacral nerve stimulator

## References

[1] Lukacz E.S., Santiago-Lastra Y., Albo M.E., Brubaker L. (2017). Urinary Incontinence in Women: A Review. JAMA.

[2] Gajewski J.B., Schurch B., Hamid R., Averbeck M., Sakakibara R., Agro E.F., Dickinson T., Payne C.K., Drake M.J., Haylen B.T. (2018). An International Continence Society (ICS) report on the terminology for adult neurogenic lower urinary tract dysfunction (ANLUTD). Neurourol. Urodyn..

[3] Fowler C.J., Griffiths D., de Groat W.C. (2008). The neural control of micturition. Nat. Rev. Neurosci..

[4] Das A.K., White M.D., Longhurst P.A. (2000). Sacral nerve stimulation for the management of voiding dysfunction. Rev. Urol..

[5] Brazzelli M., Murray A., Fraser C. (2006). Efficacy and safety of sacral nerve stimulation for urinary urge incontinence: A systematic review. J. Urol..

[6] Brusciano L., Brillantino A., Pellino G., Marinello F., Baeten C.I., Digesu A., Naldini G., Gambardella C., Lucido F.S., Sturiale A. (2023). Sacral nerve modulation for patients with fecal incontinence: Long-term outcome and effects on sexual function. Updates Surg..

[7] Liechti M.D., van der Lely S., Knupfer S.C., Abt D., Kiss B., Leitner L., Mordasini L., Tornic J., Wollner J., Mehnert U. (2022). Sacral Neuromodulation for Neurogenic Lower Urinary Tract Dysfunction. NEJM Evid..

[8] Assmann R., Douven P., Kleijnen J., van Koeveringe G.A., Joosten E.A., Melenhorst J., Breukink S.O. (2020). Stimulation Parameters for Sacral Neuromodulation on Lower Urinary Tract and Bowel Dysfunction-Related Clinical Outcome: A Systematic Review. Neuromodulation.

[9] Zeiton M., Faily S., Nicholson J., Telford K., Sharma A. (2016). Sacral nerve stimulation--hidden costs (uncovered). Int. J. Colorectal Dis..

[10] Hounsome N., Roukas C. (2018). Cost-effectiveness of sacral nerve stimulation and percutaneous tibial nerve stimulation for faecal incontinence. Ther. Adv. Gastroenterol..

[11] Jairam R., Drossaerts J., Marcelissen T., van Koeveringe G., Vrijens D., van Kerrebroeck P. (2022). Predictive Factors in Sacral Neuromodulation: A Systematic Review. Urol. Int..

[12] Gevelinger M.M., Sanderson D.J., Jaworski E., Doyle P.J. (2020). Evaluation of Sacral Nerve Stimulation Device Revision and Explantation in a Single Center, Multidisciplinary Study. Neuromodulation.

[13] Malde S., Marcelissen T., Vrijens D., Apostilidis A., Rahnama I.S., Cardozo L., Lovick T. (2020). Sacral nerve stimulation for refractory OAB and idiopathic urinary retention: Can phenotyping improve the outcome for patients: ICI-RS 2019?. Neurourol. Urodyn..

[14] Samad M., Angel M., Rinehart J., Kanomata Y., Baldi P., Cannesson M. (2023). Medical Informatics Operating Room Vitals and Events Repository (MOVER): A public-access operating room database. JAMIA Open.

[15] Akpala A., Lezama T., Jinadu K., Belal M., King T. (2024). A Five-Year Retrospective Study on the Clinical Outcomes of Sacral Nerve Stimulation for Neuromodulation of the Lower Urinary Tract in a Tertiary Hospital. Cureus.

[16] Noblett K., Siegel S., Mangel J., Griebling T.L., Sutherland S.E., Bird E.T., Comiter C., Culkin D., Bennett J., Zylstra S. (2016). Results of a prospective, multicenter study evaluating quality of life, safety, and efficacy of sacral neuromodulation at twelve months in subjects with symptoms of overactive bladder. Neurourol. Urodyn..

[17] Pedregosa F., Varoquaux G., Gramfort A., Michel V., Thirion B., Grisel O., Blondel M. (2011). Scikit-learn: Machine Learning in Python. J. Mach. Learn. Res..

[18] McKinney W. Data Structures for Statistical Computing in Python. Proceedings of the 9th Python in Science Conference.

[19] Waskom M.L. (2021). seaborn: Statistical data visualization. J. Open Source Softw..

[20] Prasada S., Desai M.Y., Saad M., Smilowitz N.R., Faulx M., Menon V., Moudgil R., Chaudhury P., Hussein A.A., Taigen T. (2022). Preoperative Atrial Fibrillation and Cardiovascular Outcomes After Noncardiac Surgery. J. Am. Coll. Cardiol..

[21] Tateosian V.S., Richman D.C. (2018). Preoperative Cardiac Evaluation for Noncardiac Surgery. Anesthesiol. Clin..

[22] Cameron A.P., Anger J.T., Madison R., Saigal C.S., Clemens J.Q. (2013). The Urologic Diseases in America Project. Battery explantation after sacral neuromodulation in the Medicare population. Neurourol. Urodyn..

[23] Myer E.N.B., Petrikovets A., Slocum P.D., Lee T.G., Carter-Brooks C.M., Noor N., Carlos D.M., Wu E., Van Eck K., Fashokun T.B. (2018). Risk factors for explantation due to infection after sacral neuromodulation: A multicenter retrospective case-control study. Am. J. Obstet. Gynecol..

