# Supplementary material for: Identification of Perioperative Risk Factors for Early Sacral Nerve Stimulator Explantation: A Single-Center Retrospective Cohort Study
Source: J Clin Med. 2025 Mar 29;14(7):2363. doi: 10.3390/jcm14072363 (PMC11989564; doi:10.3390/jcm14072363)
Supplement: Supplementary file 1 [file jcm-14-02363-s001.zip › jcm-3533832-supplementary.pdf]

Table S1: Detailed definitions of all study variables.

Variable	Definition
Age at time of implant	Age in years
Gender	Female: 1, Male: 0
SNS implant surgery	CPT: 64561, 64581, 64590<br>ICD: 00HU0MZ
SNS explantation	CPT: 64561, 64590, 64585, 64595
ASA score	American Society of Anesthesiologist Score (1-5)
Length of stay	Length of stay in days following implantation for any reason.
ICU admission	Admission to the ICU following implantation for any reason.
Past Medical History	Medical comorbidity data were queried using ICD-9 codes, with assessed diagnoses including cerebrovascular disease (ICD 429.2), failed back surgery syndrome (ICD 722.83), peripheral neuropathy (ICD 724.2), neck pain (ICD 723.1), obstructive sleep apnea (ICD 327.23), sleep disorders (ICD: 780.52, 327.23, 307.42, 327.01), hypertension (ICD 401.9), hyperlipidemia (ICD: 272.4), atrial fibrillation (ICD: 427.31), type 2 diabetes mellitus (ICD 250), chronic kidney disease (ICD: 585.9), anxiety (ICD: 300), depression (ICD: 296.2), attention deficit-hyperactivity disorder (ICD: 314.01), fibromyalgia (ICD 729.1), irritable bowel syndrome (ICD: 564.1), obesity (ICD: 278, OR Last BMI on/before SCS implant is ≥ 30), migraine (ICD: 346), urinary dysfunction (ICD 788.69), opioid use disorder (ICD: 304, 292.9, V58.83), substance use disorder (ICD: 305.9), alcohol abuse (ICD: 305, 790.3), tobacco use (ICD: V15.82, 305.1), muscle pain (ICD: 729.1), arthritis (ICD: 711, 715, 716), obsessive compulsive disorder (ICD: 300.3), post-traumatic stress disorder (ICD: 309.81) and malignancy (ICD: 199.1, 275.42).

SNS: sacral nerve stimulator

Table S2: Odds Ratio, 95% confidence interval and P-value of multivariate logistic regression after feature optimization.

Risk Factor	Odds Ratio	95% Confidence Interval	p-value
Female sex	3.72	3.68 – 3.76	<0.0001*
Age	Excluded	Excluded	N/A
Monitored anesthesia care	Excluded	Excluded	N/A
ASA score	2.39	2.36 – 2.42	<0.0001*
Length of stay	1.92	1.90 – 1.94	<0.0001*
ICU admission	Excluded	Excluded	N/A
Indication for SNS[1]			
Peripheral neuropathy	1.86	1.83 – 1.88	<0.0001*
Low back pain	1.82	1.79 – 1.84	<0.0001*
Cervical pain	Excluded	Excluded	N/A
Urinary dysfunction	Excluded	Excluded	N/A
Past Medical History			
Cerebrovascular disease	Excluded	Excluded	N/A
Obstructive sleep apnea	Excluded	Excluded	N/A
Sleep disorder	Excluded	Excluded	N/A
Hypertension	Excluded	Excluded	N/A
Hyperlipidemia	Excluded	Excluded	N/A
Atrial fibrillation	0.38	0.37 – 0.38	<0.0001*
Diabetes mellitus	Excluded	Excluded	N/A
Chronic kidney disease	0.47	0.47 – 0.48	<0.0001*
Anxiety	Excluded	Excluded	N/A
Depression	Excluded	Excluded	N/A
ADHD	Excluded	Excluded	N/A
OCD	Excluded	Excluded	N/A
PTSD	Excluded	Excluded	N/A
Fibromyalgia	Excluded	Excluded	N/A
Irritable bowel syndrome	Excluded	Excluded	N/A
Obesity	Excluded	Excluded	N/A
Migraine	Excluded	Excluded	N/A
Musculoskeletal pain	Excluded	Excluded	N/A
Arthritis	Excluded	Excluded	N/A
Malignancy	3.88	3.84 – 3.93	<0.0001
Social History			
Opioid use disorder	Excluded	Excluded	N/A
Illicit substance use	Excluded	Excluded	N/A
Alcohol use	2.08	2.06 – 2.10	<0.0001*
Tobacco products	Excluded	Excluded	N/A

[1]In patients with multiple possible indications for neuromodulation, they were included in both groups. * = $p < 0.05$. ADHD = attention deficit-hyperactivity disorder; ASA = American Society of Anesthesiologist; OCD = obsessive compulsive disorder; PTSD = post-traumatic stress disorder.
